# The Burden of Cancer and Precancerous Conditions Among Transgender Individuals in a Large Health Care Network: Retrospective Cohort Study

**DOI:** 10.2196/73843

**Published:** 2025-09-08

**Authors:** Shuang Yang, Yongqiu Li, Christopher W Wheldon, Jessica Y Islam, Mattia Prosperi, Thomas J George, Elizabeth A Shenkman, Fei Wang, Jiang Bian, Yi Guo

**Affiliations:** 1 Department of Health Outcomes and Biomedical Informatics, University of Florida, 1889 Museum Road, Suite 7000, Gainesville, FL, 32611, United States, 1 352 294-5969; 2 Department of Social and Behavioral Sciences, Temple University, Philadelphia, PA, United States; 3 Department of Cancer Epidemiology, Moffitt Cancer Center, Tampa, FL, United States; 4 Department of Epidemiology, Unvirsity of Florida, Gainesville, FL, United States; 5 Department of Medicine, University of Florida, Gainesville, FL, United States; 6 Department of Population Health Sciences, Weill Cornell Medicine, New York, NY, United States

**Keywords:** transgender and gender nonconforming, cancer cumulative incidence, real-world data, electronic health records, health disparities

## Abstract

**Background:**

Disparities in cancer burden between transgender and cisgender individuals remain an underexplored area of research.

**Objective:**

This study aimed to examine the cumulative incidence and associated risk factors for cancer and precancerous conditions among transgender individuals compared with matched cisgender individuals.

**Methods:**

We conducted a retrospective cohort study using patient-level electronic health record (EHR) data from the University of Florida Health Integrated Data Repository between 2012 and 2023. Transgender individuals were identified using a validated, computable phenotype algorithm that used structured data and clinical notes. They matched 1:10:10 by age and calendar year of index date with cisgender women and cisgender men. The index date was the first transgender-related record for transgender individuals and a matched diagnosis date for cisgender controls. Primary outcomes included new-onset cancers associated with human papillomavirus, human immunodeficiency virus, tobacco, alcohol, lung, breast, and colorectal sites. Secondary outcomes were precancerous conditions related to the same cancer types. We calculated cumulative incidence rates and conducted time-to-event analyses using the Fine-Gray method, treating all-cause death as a competing risk, to assess associations between gender identity and the presence of cancer or precancer, adjusting for demographic and clinical covariates. Interaction analyses evaluated if associations between cancer risk factors and precancer differed by gender identity.

**Results:**

We identified 2745 transgender individuals (mean age at index date 25.1, SD 14.0 years) and matched them with 27,450 cisgender women and 27,450 cisgender men from the same health care system. The cumulative incidence of cancer did not differ significantly between transgender and cisgender cohorts (transgender n=28, 1.0% vs cisgender women, n=358, 1.3%; *P*=.13 and cisgender men, n=314, 1.1%; *P*=.64). However, transgender individuals exhibited significantly higher risks for precancerous conditions compared to cisgender women (subdistribution hazard ratios [sHRs] 1.1, 95% CI 1.0‐1.3) and cisgender men (sHR 1.3; 95% CI 1.2‐1.5). Specifically, transgender individuals were more likely to develop colorectal precancer (sHR 1.2; 95% CI 1.1‐1.4) compared to cisgender women, as well as human papillomavirus–related precancer (sHR 1.8; 95% CI 1.4‐2.3) and colorectal precancer (sHR 1.4; 95% CI 1.2‐1.6) compared to cisgender men. Subgroup analyses showed similar patterns in both female-to-male and male-to-female individuals compared with their matched cisgender counterparts. Interaction analyses revealed stronger protective effects of private insurance or Medicare against precancers in transgender individuals than in cisgender peers, while being non-Hispanic Black or having substantial comorbidities were stronger risk factors among transgender individuals.

**Conclusions:**

Transgender individuals showed a similar cancer incidence yet significantly higher precancer incidence compared with cisgender individuals, suggesting underdiagnosis or delayed detection. These findings highlight the need for tailored preventive care strategies, including targeted screenings and risk reduction interventions, to address cancer disparities in the transgender population.

## Introduction

Transgender persons are individuals whose gender identity or expression does not align with society’s expectations based on the sex assigned at birth [1]. Global estimates suggest that 0.3%‐0.5% of adults self-identify as transgender [2], and a recent survey reported that as many as 1.6% of US adults identify as transgender [3]. Transgender individuals consistently experience a disproportionately high prevalence of adverse health outcomes, including human immunodeficiency virus (HIV), sexually transmitted infections, mental health distress, and substance use disorders [4]. Despite the growing literature on the unique health challenges faced by transgender individuals, our understanding of the cancer burden (eg, cumulative incidence of cancer) and risk in this population remains limited [5], primarily due to the lack of relevant data [6]. Reluctance to self-identify and participate in health surveys, along with the limited inclusion of gender identity measures in national surveillance systems, poses challenges in obtaining population-based representative samples and conducting longitudinal studies to examine cancer burden in this population. Current insights into cancer risk in transgender populations rely primarily on anecdotal evidence and small-scale studies [7,8], leading to inconsistent findings.

Evidence suggests that transgender individuals may face an elevated risk of certain cancers compared to the general population, including breast cancer [7], cancers linked to human papillomavirus (HPV) [9] and HIV [6], and those associated with tobacco and alcohol use [10,11]. For example, transgender women (male-to-female, MTF) undergoing prolonged hormone therapy are exposed to high estrogen levels, which may increase breast cancer risk [12-14]. Transgender populations are twice as likely to smoke as cisgender peers [15] and have higher rates of hazardous alcohol use [16], both known cancer risk factors. In addition, a higher prevalence of sexual health risk behaviors [17-20] increases the risk of acquiring cancer-related infections, including HPV and HIV. Studies have reported a higher cervicovaginal HPV prevalence among transgender individuals assigned female at birth compared to cisgender women (30.5% vs 20.0%) [17] and a global HIV prevalence of 19.9% for trans women and 2.6% for trans men, which are 66 and 6.8 times higher than the general population, respectively [18]. These infections further elevate the risk of virus-associated cancers.

However, due to disparities in health care access and screening, cancer is often diagnosed at later stages or underreported in transgender populations. Studies have shown that transgender men are less likely to be up to date on Pap tests compared to cisgender women, and transgender individuals overall have lower rates of cervical cancer screening [19,20]. These disparities are attributed to fear of discrimination in health care settings, lack of provider knowledge about transgender health needs, and the absence of inclusive screening guidelines [21-24]. As a result, transgender individuals frequently experience delayed diagnosis or remain undiagnosed. These challenges contribute to late-stage diagnoses, poorer health outcomes, and an underestimation of the true cancer burden in this population.

Given these challenges, investigating precancerous lesions offers a timely and meaningful opportunity to address cancer disparities. Precancerous lesions, or precancers, refer to abnormal cellular and tissue changes that have the potential to develop into cancer if left untreated [25]. The progression rates of these lesions vary depending on their type and location. For instance, cervical intraepithelial neoplasia, a common HPV-related precancerous condition, is a critical early indicator of cervical cancer risk [26]. Previous studies have shown that cervical intraepithelial neoplasia 3 is associated with a twofold increased risk of cervical cancer compared to the general female population [27]. Investigating the burden of precancer in transgender individuals is crucial for several reasons. First, many transgender individuals are relatively young, and while their cumulative cancer incidence may be low, a significant proportion may already harbor precancerous lesions, signaling increased future cancer risk. Second, studying precancer can reveal disparities missed by cancer diagnoses alone, especially in underserved populations. Even when detection is limited, observed lesions highlight underlying vulnerability and offer earlier opportunities for intervention. Thus, examining the cumulative incidence and risk factors for precancer among transgender individuals can uncover hidden disparities, inform targeted prevention strategies, and help reduce future cancer burden in this population.

Electronic health record (EHR) data have emerged as a vital tool for addressing knowledge gaps in cancer research within the transgender community. The widespread adoption of EHRs enables large-scale, longitudinal studies and comparisons with cisgender controls. EHRs offer both structured data (eg, diagnoses and procedures) and unstructured clinical notes, which can help identify transgender individuals and capture health profiles more comprehensively. Unlike traditional cancer registries, EHRs include detailed risk factor data, allowing for a more nuanced understanding of disparities. In addition, their broad population coverage allows for robust comparisons between transgender and cisgender individuals. In this study, we leveraged longitudinal EHR data to examine the cumulative incidence and risk factors for cancer and precancer by comparing transgender and cisgender individuals. We hypothesized that transgender individuals would have a heightened risk for cancer and precancer compared to cisgender individuals.

## Methods

### Data Source and Study Population

We conducted a retrospective cohort study using patient-level EHR data from 2012‐2023, obtained from the University of Florida (UF) Health Integrated Data Repository (IDR). This clinical data warehouse aggregates data from UF’s various clinical and administrative information systems, including the Epic EHR system. The IDR contains more than 1 billion observational facts for over 2 million patients. This study followed the STROBE (Strengthening the Reporting of Observational Studies in Epidemiology) and RECORD (Reporting of Studies Conducted using Observational Routinely-Collected Data) guidelines (STROBE-RECORD; Checklist 1).

The study population included a cohort of transgender individuals and a matched cohort of cisgender individuals identified in the UF Health EHRs. Transgender individuals and their sex assigned at birth were identified using a previously developed and validated computable phenotype algorithm [28]. The algorithm classified individuals as transgender if they met at least one of the following criteria: (1) recorded gender identity as transgender, or (2) had at least one transgender diagnosis code from the diagnosis table, along with at least one transgender keyword (eg, “transgender,” “MTF,” and “FTM”) from clinical notes. The complete list of keywords and codes is provided in Multimedia Appendix 1. Manual chart review of 300 records demonstrated high algorithm performance: precision=0.96, recall=0.95, *F*_1_-score=0.96. In an independent test set, the algorithm achieved perfect precision and recall (*F*_1_-score=1.0). We further classified transgender individuals into female-to-male (FTM), MTF, or unknown categories based on sex assigned at birth.

The index date for transgender individuals was defined as the date of the first recorded evidence of transgender status. Each transgender individual was matched to 10 cisgender men and 10 cisgender women, randomly selected from the IDR, based on age and calendar year of the index date. This dual matching approach enables comparisons with both cisgender groups to isolate disparities related to gender identity versus sex assigned at birth. Similar matching approaches have been used in prior transgender health research [29,30]. The index date for cisgender individuals was a randomly selected date of diagnosis. We excluded individuals who (1) had an age at first encounter of less than 3 years, (2) had no encounter after the index date, and (3) had a gap of longer than 2 years between any two encounters.

### Study Outcomes

Primary outcomes were new-onset cancer cases between 2012 and 2023, assessed from the earliest available record until the date of cancer occurrence, loss to follow-up, or study termination. Cancer types included HPV-related, HIV-related, tobacco-related, and alcohol-related cancers [31-34], as well as lung, breast, and colorectal cancers, given the existence of clear screening guidelines for these types. Specifically, HPV-related cancers included oropharyngeal, cervical, anal, vaginal, vulvar, and penile cancers [31]. HIV-related cancers included Kaposi sarcoma, non-Hodgkin lymphoma, and cervical cancer [32]. Tobacco-related cancers encompassed lung, head and neck, liver, esophagus, bladder, kidney, stomach, pancreas, colorectal, and cervical cancers [34], while alcohol-related cancers comprised head and neck, esophageal, liver, breast, and colorectal cancers [33]. We also calculated cases of “any cancer,” covering all 18 individual cancer sites mentioned above. Patients with these cancers were identified using *International Classification of Diseases (ICD)−9/10 CM* diagnosis codes from the UF Health EHRs.

Secondary outcomes were new-onset precancerous conditions for the same 18 cancer types, defined using criteria from the Canadian Cancer Society [25] and prior literature [35-42]. Similar to the cancer outcomes, we grouped these precancerous conditions into HPV-related precancer, tobacco-related precancer, alcohol-related precancer, prelung cancer, prebreast cancer, and precolorectal cancer categories. We curated ICD-9/10 codes from established sources and clinical review (Multimedia Appendix 2) to harmonize definitions and minimize misclassification, especially for less frequently documented conditions.

### Covariates

Covariates included age at the index date, race-ethnicity, insurance payer, smoking status, family history of cancer, health care usage (ie, outpatient and inpatient visits), and Charlson Comorbidity Index (CCI). Race-ethnicity was categorized as non-Hispanic White (NHW), non-Hispanic Black (NHB), non-Hispanic Other, Hispanic, or Unknown. Insurance payers were categorized as Medicare, private insurance, self-pay, or Medicaid or other insurance (eg, charity and worker’s compensation). Smoking status was determined from the most recent EHR entry before the index date and categorized as current, former, never smoker, or unknown. In addition, baseline measures of health care usage, family cancer history (ICD-9: V16 and ICD-10: Z80), and CCI were extracted from the EHR data within 12 months before the index date. Health care usage was measured based on the number of outpatient and inpatient visits. Family history of cancer and conditions in the CCI (eg, diabetes) were identified using relevant ICD-9/10-CM codes and confirmed with at least one inpatient or outpatient diagnosis. We calculated the CCI using the modified algorithm by Klabunde et al [43]. CCI was categorized into 3 groups: no comorbidity (CCI=0), some comorbidities (CCI=1), or substantial burden of comorbidities (CCI ≥2). For variables with missing values (eg, smoking status and insurance payer), we created an “unknown” category. We included it in both univariate comparisons and regression models to retain the full analytic sample. No other variables had missing values.

### Statistical Analysis

We summarized baseline characteristics by gender identity and transgender subgroups. We calculated and compared cumulative incidence rates of cancer and precancerous conditions between transgender individuals and their matched cisgender male and female counterparts. Given the cohort’s relatively young age and low baseline disease probability, cumulative incidence was an appropriate measure, reflecting new cases during follow-up as a proportion of the population at risk. Means (SDs) or medians (IQRs) were used for continuous variables. Categorical variables were summarized with counts and percentages. Normality was assessed using the Kolmogorov-Smirnov test. Between-group comparisons used *t* tests, Wilcoxon signed-rank tests, and chi-square or Fisher exact tests, as appropriate. To estimate the hazard of each cancer and precancer outcome, we used Fine-Gray competing risk models, treating all-cause death as a competing risk. Both univariable and multivariable models were constructed, adjusting for all covariates. Adjusted subdistribution hazard ratios (sHRs) with 95% CIs were reported. Proportional hazard assumptions were verified using supremum tests. Interaction terms between gender identity and covariates were tested to evaluate differential effects. Subgroup analyses compared FTM individuals to cisgender women and MTF individuals to cisgender men using the originally matched cohort, without additional rematching to preserve statistical power and comparability. All tests were 2-sided with *α*=.05. Data processing was conducted in Python (version 3.9.4; Python Software Foundation), and statistical analyses were performed in SAS (version 9.4; SAS Institute Inc).

### Ethical Considerations

This study was approved by the UF Institutional Review Board (IRB202100946). It used deidentified EHR data, exempting it from informed consent under institutional policy [44]. Sensitive gender identity data were stored and analyzed on secure servers with access limited to authorized study personnel. No identifiable personal information was included in the analytic dataset to ensure confidentiality and compliance with ethical standards. No participant compensation was provided.

## Results

We identified 2745 transgender individuals, matched to 27,450 cisgender women and 27,450 cisgender men from the UF Health EHRs in a 1:10:10 matching ratio based on age and calendar year of the index date. Table 1 presents the distributions of patient characteristics stratified by gender identity. The mean age at index was 25.1 (SD 14.0) years across all three cohorts (*P≥*.99). Despite age matching, significant differences were observed in all other characteristics. Compared to cisgender individuals, transgender individuals were more likely to be NHW (transgender, n=1803, 65.7% vs women, n=14675, 53.5% and men, n=15,307, 55.8%; *P*<.001) and to have a substantial burden of comorbidities (transgender, n=191, 7.0% vs women, n=1148, 4.2% and men, n=1281, 4.7%, respectively; *P*<.001). In contrast, the transgender individuals were less likely to have private insurance (transgender, n=1147, 41.8% vs women, n=13,948, 50.8% and men, n=13,837, 50.4%, respectively; *P*<.001), to be never smokers (transgender, n=1569, 57.2% vs women, n=19656, 71.6% and men, n=17,330, 63.1%, respectively; *P*<.001), and to have outpatient visits [(mean 3.2, SD 6.1 vs women, mean 4.3, SD 6.8 and men, mean 3.5, SD 6.9) visits in the past year, respectively; *P*<.001]. Furthermore, transgender individuals were less likely to have a family cancer history than cisgender women (n=19, 0.7% vs n=416, 1.5%; *P*<.001) and had fewer inpatient visits in the past year than cisgender men (mean 0.15, SD 0.6 vs mean 0.2, SD 0.6; *P*<.001). Among the 2745 transgender individuals, 1324 (48.2%) were FTM, 1168 (42.6%) were MTF, and the remaining 253 (9.2%) had unknown natal sex (Table 2). Compared with MTF individuals, FTM individuals were younger [mean 22.4 (SD 11) vs mean 27.7 (SD 15.5) years; *P*<.001], more likely to be NHW (n=936, 70.7% vs n=752, 64.4%; *P*<.001), had higher rates of private insurance (n=600, 45.3% vs n=489, 41.9%; *P*<.001), were more likely to be nonsmokers (n=814, 61.5% vs n=673, 57.6%; *P*<.001), and had more outpatient visits (mean 3.4, SD 6.6 vs 3.3, SD 6.5; *P*=.004).

**Table 1.** Distributions of demographic characteristics by gender identity (N=57,645).

Variable	Transgender (n=2745)	Women^a^ (n=27,450)	*P* value	Men^a^ (n=27,450)	*P* value
Age at index date, mean (SD)	25.1 (14.0)	25.1 (14.0)	≥.99	25.1 (14.0)	≥.99
Race-ethnicity, n (%)			<.001		<.001
NHW^b^	1803 (65.7)	14,675 (53.5)		15,307 (55.8)	
NHB^c^	284 (10.4)	6344 (23.1)		5859 (21.3)	
Non-Hispanic Other	181 (6.6)	1925 (7.0)		1882 (6.9)	
Hispanic individuals	242 (8.8)	3119 (11.4)		2699 (9.8)	
Unknown	235 (8.6)	1387 (5.1)		1703 (6.2)	
Insurance payer, n (%)			<.001		<.001
Private	1147 (41.8)	13,948 (50.8)		13,837 (50.4)	
Medicaid and others^d^	539 (19.6)	9708 (35.4)		8772 (32.0)	
Medicare	181 (6.6)	1261 (4.6)		1351 (4.9)	
Self-pay	132 (4.8)	1516 (5.5)		2292 (8.4)	
Unknown	746 (27.2)	1017 (3.7)		1198 (4.4)	
Smoking status, n (%)			<.001		<.001
Current smoker	289 (10.5)	1792 (6.5)		2932 (10.7)	
Former smoker	302 (11.0)	2196 (8.0)		2409 (8.8)	
Never smoker	1569 (57.2)	19,656 (71.6)		17,330 (63.1)	
Unknown	585 (21.3)	3806 (13.9)		4779 (17.4)	
Family cancer history, n (%)			<.001		.75
No	2726 (99.3)	27,034 (98.5)		27,245 (99.2)	
Yes	19 (0.7)	416 (1.5)		205 (0.8)	
CCI,^e^ n (%)			<.001		<.001
0	2154 (78.5)	22,321 (81.3)		21,935 (79.9)	
1	400 (14.6)	3981 (14.5)		4234 (15.5)	
≥2	191 (7.0)	1148 (4.2)		1281 (4.7)	
Number of outpatient visits, mean (SD)	3.2 (6.1)	4.3 (6.8)	<.001	3.5 (6.9)	.05
Number of inpatient visits, mean (SD)	0.15 (0.6)	0.15 (0.5)	.50	0.2 (0.6)	<.001

a 1:10 matched with transgender individuals.

b NHW: non-Hispanic White.

c NHB: non-Hispanic Black.

d Other insurance includes charity and worker’s compensation.

e CCI: Charlson Comorbidity Index (0: no comorbidity; 1: some comorbidities; ≥2: substantial burden of comorbidities).

**Table 2.** Distributions of demographic characteristics by transgender subgroups (n=2745).

Variable	Transgender (N=2745)	FTM^a^ (n=1324)	MTF^b^ (n=1168)	Unknown (n=253)	*P* value
Age at index date, mean (SD)	25.1 (14.0)	22.4 (11.0)	27.7 (15.5)	26.8 (17.7)	<.001
Race-ethnicity, n (%)					<.001
NHW^c^	1803 (65.7)	936 (70.7)	752 (64.4)	115 (45.5)	
NHB^d^	284 (10.4)	105 (7.9)	160 (13.7)	19 (7.5)	
Non-Hispanic Other	181 (6.6)	78 (5.9)	90 (7.7)	13 (5.1)	
Hispanics	242 (8.8)	130 (9.8)	94 (8.1)	18 (7.1)	
Unknown	235 (8.6)	75 (5.7)	72 (6.2)	88 (34.8)	
Insurance payer, n (%)					<.001
Private	1147 (41.8)	600 (45.3)	489 (41.9)	58 (22.9)	
Medicaid and others^e^	539 (19.6)	267 (20.2)	243 (20.8)	29 (11.5)	
Medicare	181 (6.6)	68 (5.1)	105 (9.0)	8 (3.2)	
Self-pay	132 (4.8)	58 (4.4)	66 (5.7)	8 (3.2)	
Unknown	746 (27.2)	331 (25.0)	265 (22.7)	150 (59.3)	
Smoking status, n (%)					<.001
Current smoker	289 (10.5)	117 (8.8)	153 (13.1)	19 (7.5)	
Former smoker	302 (11.0)	149 (11.3)	142 (12.2)	11 (4.4)	
Never smoker	1569 (57.2)	814 (61.5)	673 (57.6)	82 (32.4)	
Unknown	585 (21.3)	244 (18.4)	200 (17.1)	141 (55.7)	
Family cancer history, n (%)					.82
No	2726 (99.3)	1314 (99.2)	1160 (99.3)	252 (99.6)	
Yes	19 (0.7)	10 (0.8)	8 (0.7)	1 (0.4)	
CCI^f^, n (%)					<.001
0	2154 (78.5)	1065 (80.4)	852 (73.0)	237 (93.7)	
1	400 (14.6)	197 (14.9)	193 (16.5)	10 (4.0)	
≥2	191 (7.0)	62 (4.7)	123 (10.5)	6 (2.4)	
Number of outpatient visits, mean (SD)	3.2 (6.1)	3.4 (6.6)	3.3 (6.5)	1.8 (3.8)	.004
Number of inpatient visits, mean (SD)	0.15 (0.6)	0.16 (0.6)	0.15 (0.7)	0.06 (0.2)	.07

a FTM: female-to-male.

b MTF: male-to-female.

c NHW: non-Hispanic White.

d NHB: non-Hispanic Black.

e Other insurance: charity and worker’s compensation.

f CCI: Charlson Comorbidity Index (0: no comorbidity; 1: some comorbidities; ≥2: substantial burden of comorbidities).

Median follow-up time for cancer was 1176 days (IQR 344‐2579) in the transgender group, 1227 days (IQR 379‐2669) for cisgender women, and 999 days (IQR 232‐2406) for cisgender men. For precancer, the median follow-up was 1031 days (IQR 307‐2289) for transgender individuals, 1090 days (IQR 321‐2425) for cisgender women, and 931 days (IQR 199‐2230) for cisgender men. Table 3 shows that the cumulative incidence of any cancer was similar between transgender individuals (n=28, 1.0%) and cisgender women (n=358, 1.3%; *P*=.13) and men (n=314, 1.1%; *P*=.64). The only significant difference was a higher rate of breast cancer in transgender individuals compared with cisgender men (n=7, 0.3% vs n=1, 0.004%; *P*<.001). For precancer cases, the cumulative incidence of any precancer was higher in transgender individuals (n=306, 11.1%) than in cisgender men (n=2758, 10.0%; *P*=.07) but similar to cisgender women (n=3086, 11.2%; *P*=.88), although these comparisons were not statistically significant. Compared with cisgender men, transgender individuals had a significantly higher cumulative incidence of HPV-related precancers (n=76, 2.8% vs n=413, 1.5%; *P*<.001), prebreast cancer (n=2, 0.07% vs n=1, 0.01%; *P*=.02), and precolorectal cancer (n=184, 6.7% vs n=1472, 5.4%; *P*=.004). However, precancer incidence did not differ significantly between transgender individuals and cisgender women.

Table 4 shows subgroup results. Compared to cisgender women, FTM individuals had significantly lower cumulative incidence of any cancer (n=9, 0.7% vs n=358, 1.3%; *P*=.05) and any precancer (n=123, 9.3% vs n=3086, 11.2%; *P*=.03), including lower HPV-related (n=21, 1.6% vs n=877, 3.2%; *P*=.001) and alcohol-related precancers (1.1% vs 2.3%; *P*=.003). These lower rates may reflect reduced smoking and alcohol consumption in FTM individuals, as well as a reduced risk of HPV-related gynecologic cancers due to lower gynecologic health care engagement or differences in screening practices. In contrast, MTF individuals largely followed the overall transgender versus cisgender men trends, but with additional differences. They had a higher cumulative incidence of breast cancer (n=2, 0.2% vs n=1, 0.004%; *P*=.005), any precancer (n=171, 14.6% vs n=2758, 10.0%; *P*<.001), HPV-related precancers (n=53, 4.5% vs n=413, 1.5%; *P*<.001), and precolorectal cancer (n=99, 8.5% vs n=1472, 5.4%; *P*=.004), consistent with the overall transgender cohort. However, unlike the broader transgender comparison, MTF individuals also had higher cumulative incidence of tobacco-related (n=144, 12.3% vs n=2462, 9.0%; *P*<.001) and alcohol-related precancer (n=38, 3.3% vs n=599, 2.2%; *P*=.02), while prebreast cancer rates did not differ from cisgender men (n=0, 0.0% vs n=1, 0.01%; *P*≥.99).

**Table 3.** Cumulative incidence of cancer and precancer cases by gender identity (N=57,645).

Condition	Transgender (n=2745), n (%)	Women (n=27,450), n (%)	*P* value	Men (n=27,450), n (%)	*P* value
Cancer					
Any cancer^a^	28 (1.0)	358 (1.3)	.13	314 (1.1)	.64
HPV-related^b^	10 (0.4)	69 (0.3)	.24	69 (0.3)	.24
HIV-related^c^	8 (0.3)	92 (0.3)	.70	87 (0.3)	≥.99
Tobacco-related^d^	15 (0.6)	168 (0.6)	.80	219 (0.8)	.17
Alcohol-related^e^	12 (0.4)	185 (0.7)	.17	116 (0.4)	.88
Lung	9 (0.3)	53 (0.2)	.18	81 (0.3)	.71
Breast	7 (0.3)	145 (0.5)	.06	1 (0.004)	<.001
Colorectal	4 (0.2)	45 (0.2)	≥.99	41 (0.2)	≥.99
Precancer					
Any precancer^a^	306 (11.1)	3086 (11.2)	.88	2758 (10.0)	.07
HPV-related^b^	76 (2.8)	877 (3.2)	.22	413 (1.5)	<.001
Tobacco-related^d^	272 (9.9)	2850 (10.4)	.44	2462 (9.0)	.10
Alcohol-related^e^	55 (2.0)	629 (2.3)	.33	599 (2.2)	.54
Lung	9 (0.3)	79 (0.3)	.71	129 (0.5)	.29
Breast	2 (0.07)	56 (0.2)	.17	1 (0.01)	.02
Colorectal	184 (6.7)	1728 (6.3)	.41	1472 (5.4)	.004

a Any of the 18 cancer types: Kaposi sarcoma, non-Hodgkin lymphoma, cervical cancer, oropharyngeal, anal, vaginal, vulvar, penile, lung, head and neck, liver, esophagus, bladder, kidney, stomach, pancreas, colorectal, and breast cancer.

b HPV-related cancers include oropharyngeal, cervical, anal, vaginal, vulvar, and penile cancers.

c HIV-related cancer includes Kaposi sarcoma, non-Hodgkin lymphoma, and cervical cancer.

d Tobacco-related cancers include lung, head and neck, liver, esophagus, bladder, kidney, stomach, pancreas, colorectal, and cervical cancers.

e Alcohol-related cancers include head and neck, esophageal, liver, breast, and colorectal cancers.

**Table 4.** Cumulative incidence of cancer cases by transgender subgroups (n=57,392).

Condition	FTM^a^ (n=1324), n (%)	Women (n=27,450), n (%)	*P* value	MTF^b^ (n=1168), n (%)	Men (n=27,450), n (%)	*P* value
Cancer						
Any cancer^c^	9 (0.7)	358 (1.3)	.05	17 (1.5)	314 (1.1)	.33
HPV-related^d^	5 (0.3)	69 (0.3)	.38	4 (0.3)	69 (0.3)	.55
HIV-related^e^	4 (0.3)	92 (0.3)	≥.99	4 (0.3)	87 (0.3)	.79
Tobacco-related^d^	5 (0.4)	168 (0.6)	.28	8 (0.7)	219 (0.8)	.67
Alcohol-related^f^	6 (0.4)	185 (0.7)	.33	5 (0.4)	116 (0.4)	.98
Lung	1 (0.08)	53 (0.2)	.52	7 (0.6)	81 (0.3)	.07
Breast	5 (0.4)	145 (0.5)	.46	2 (0.2)	1 (0.004)	.005
Colorectal	1 (0.08)	45 (0.2)	.72	3 (0.3)	41 (0.2)	.43
Precancer						
Any precancer^c^	123 (9.3)	3086 (11.2)	.03	171 (14.6)	2758 (10.0)	<.001
HPV-related^d^	21 (1.6)	877 (3.2)	.001	53 (4.5)	413 (1.5)	<.001
Tobacco-related^d^	116 (8.8)	2850 (10.4)	.06	144 (12.3)	2462 (9.0)	<.001
Alcohol-related^f^	14 (1.1)	629 (2.3)	.003	38 (3.3)	599 (2.2)	.02
Lung	3 (0.2)	79 (0.3)	≥.99	6 (0.5)	129 (0.5)	.83
Breast	2 (0.2)	56 (0.2)	≥.99	0 (0.0)	1 (0.01)	≥.99
Colorectal	78 (5.9)	1728 (6.3)	.55	99 (8.5)	1472 (5.4)	.004

a FTM: female-to-male.

b MTF: male-to-female.

c Any of the 18 cancer types: Kaposi sarcoma, non-Hodgkin lymphoma, cervical cancer, oropharyngeal, anal, vaginal, vulvar, penile, lung, head and neck, liver, esophagus, bladder, kidney, stomach, pancreas, colorectal, and breast cancer.

d HPV-related cancers include oropharyngeal, cervical, anal, vaginal, vulvar, and penile cancers.

e HIV-cancer includes Kaposi sarcoma, non-Hodgkin lymphoma, and cervical cancer. Tobacco-related cancers include lung, head and neck, liver, esophagus, bladder, kidney, stomach, pancreas, colorectal, and cervical cancers.

f Alcohol-related cancers include head and neck, esophageal, liver, breast, and colorectal cancers.

**Figure 1.**
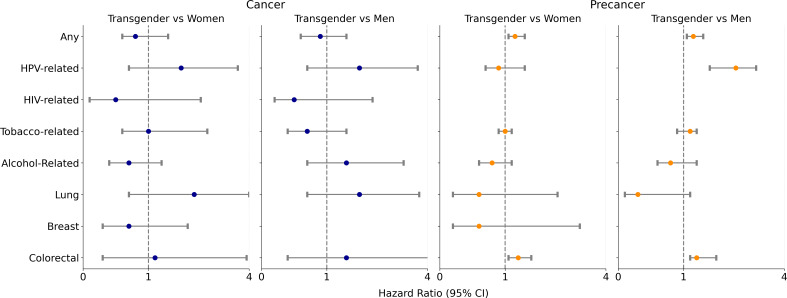
Adjusted hazard ratios estimating the association of gender identity with cancer and precancer cases. HIV: human immunodeficiency virus; HPV: human papillomavirus.

Figure 1 summarizes the main results from the Fine-Gray regression examining associations between gender identity and cancer or precancer outcomes. For cancer cases, the risk of developing any cancer or individual cancer types was statistically the same across the transgender and cisgender cohorts; none of the adjusted sHRs for gender identity were statistically significant after adjusting for covariates. For precancer cases, transgender individuals were significantly more likely to develop any precancer than cisgender women (adjusted sHR 1.1, 95% CI 1.0‐1.3) and men (adjusted sHR 1.3, 95% CI 1.2‐1.5). Compared with cisgender women, transgender individuals were more likely to develop precolorectal cancer (adjusted sHR 1.2, 95% CI 1.1‐1.4). Compared with cisgender men, transgender individuals were more likely to develop HPV-related precancers (adjusted sHR 1.8, 95% CI 1.4‐2.3) and precolorectal cancer (adjusted sHR 1.4, 95% CI 1.2‐1.6) after adjusting for covariates.

**Figure 2.**
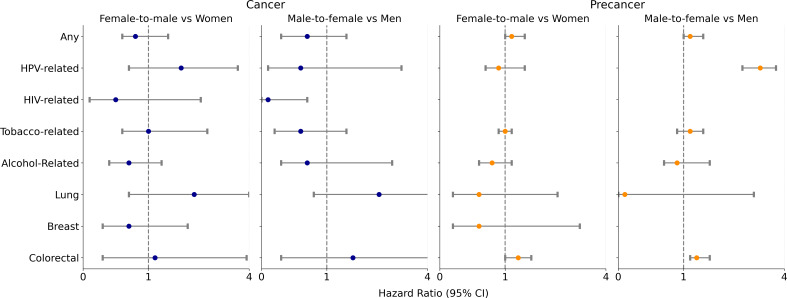
Adjusted hazard ratios estimating the association of subtype gender identity with cancer and precancer cases. HIV: human immunodeficiency virus; HPV: human papillomavirus.

The findings from the multivariable regression using the Fine-Gray method for subgroups are summarized in Figure 2. FTM individuals were significantly more likely to develop any precancer than cisgender women (adjusted sHR 1.1, 95% CI 1.0‐1.3), and MTF individuals were significantly more likely to develop any precancer than cisgender men (adjusted sHR 1.5, 95% CI 1.2‐1.8). Compared with cisgender women, FTM individuals were more likely to develop precolorectal cancer (adjusted sHR 1.2, 95% CI 1.0‐1.4). Compared with cisgender men, MTF individuals were more likely to develop HPV-related precancers (adjusted sHR 2.5, 95% CI 1.9‐3.4) and precolorectal cancer (adjusted sHR 1.6, 95% CI 1.3‐1.9) after adjusting for covariates.

**Figure 3.**
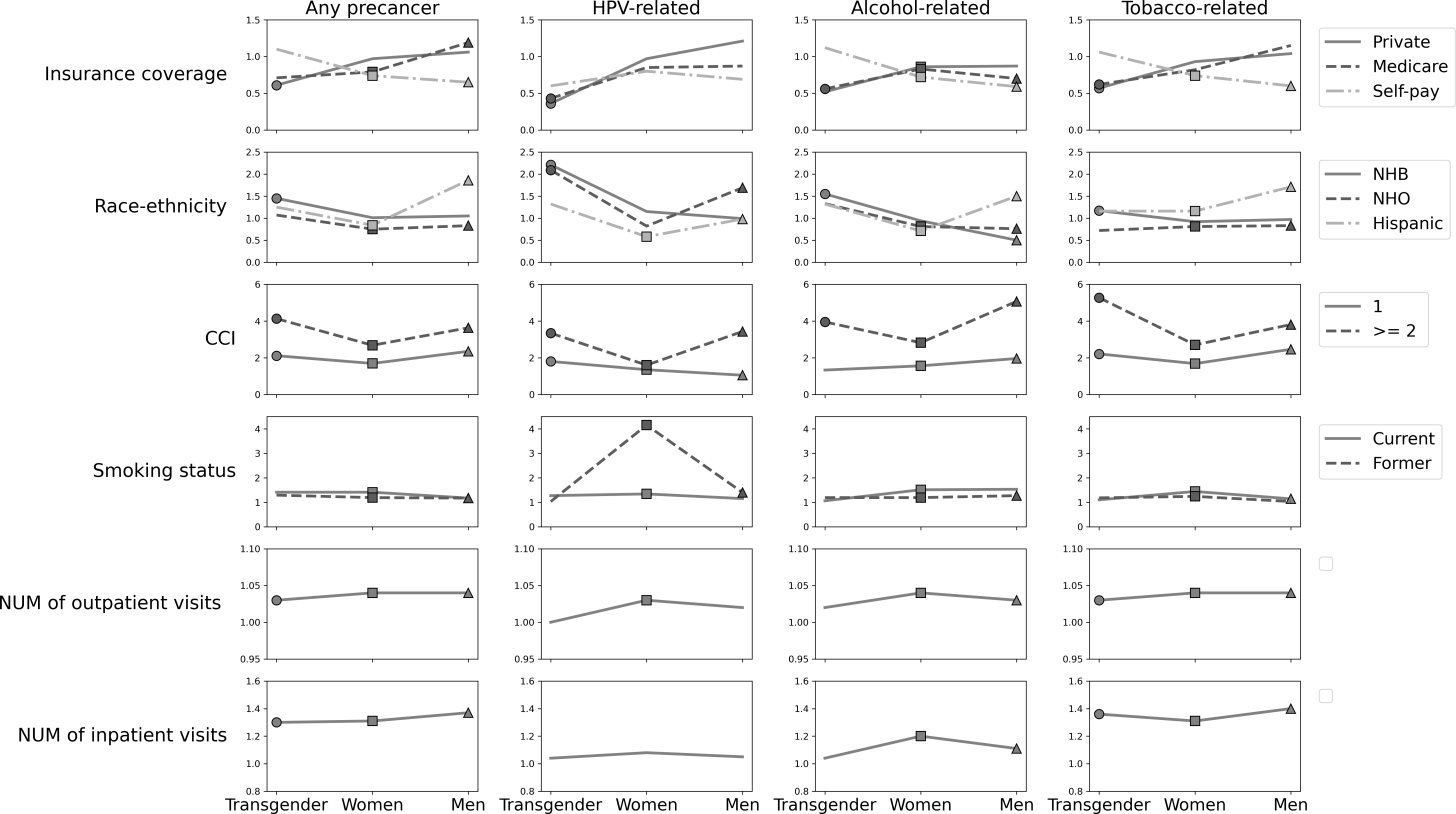
Interaction plots of gender identity with cancer risk factors for precancer diseases. CCI: Charlson Comorbidity Index (0: no comorbidity, 1: some comorbidities, and ≥2: substantial burden of comorbidities); HPV: human papillomavirus; NHB: non-Hispanic Black; NHO: non-Hispanic Other; NUM: number.

We assessed whether associations between cancer risk factors and precancer risk varied by gender identity using interaction analysis (Figure 3). Each x-axis position represents a gender group (transgender, cisgender women, and cisgender men), and each line represents one category of a risk factor (eg, insurance type, race, and smoking), labeled at the right. Markers (dots, squares, and triangles) indicate significant associations (*P*<.05), sHRs >1 indicate increased risk, and sHRs <1 indicate a protective effect. Interaction effects are identified by changes in sHRs across gender groups. For insurance coverage, having private insurance or Medicare was a protective factor against precancers, with a greater protective effect in transgender individuals than in cisgender individuals. Being NHB, compared with NHW, was a significant risk factor for any precancer in transgender individuals. For all precancer types analyzed, being NHB was a greater risk factor for transgender individuals than for cisgender individuals. Hispanic ethnicity was a significant risk factor for precancers only in cisgender individuals, with the risk being higher in men than in women. Having a substantial burden of comorbidities (CCI ≥2 vs CCI=0) was a greater risk factor for transgender individuals for any precancer and tobacco-related precancer. Regarding smoking status, being a former smoker, compared with a never smoker, was a significant risk factor for HPV-related precancers in cisgender individuals, with the risk being higher in women than in men. The effects of outpatient and inpatient visits on precancer risk were smaller in transgender individuals than in cisgender individuals.

X-axis position represents a gender group (transgender, cisgender women, and cisgender men). The y-axis represents sHRs values. Each line represents one category of a risk factor. The dots, squares, and triangles represent statistically significant sHRs for transgender, cisgender women, and cisgender men, respectively. The reference groups are Medicaid for insurance coverage, NHW for race-ethnicity, 0 for CCI, and never smoker for smoking status.

## Discussion

### Principal Findings

In this study, we used EHR data from a large health care system to examine the risk of cancer and precancer and explore the impact of cancer risk factors across different gender identities. Our findings indicate that while the hazard of developing any cancer was similar between transgender and cisgender individuals, transgender individuals were more likely to develop precancer conditions than their cisgender counterparts. Specifically, transgender individuals showed elevated risks for colorectal precancers compared with cisgender women, and higher risks for HPV-related and colorectal precancers compared with cisgender men in the time-to-event analysis. These associations remained significant in MTF individuals compared with cisgender men and in FTM individuals compared with cisgender women after adjustment. Furthermore, our analysis identified private insurance or Medicare as protective factors against precancers in transgender individuals, while factors such as being NHB or having a substantial burden of comorbidities emerged as significant risk factors.

### Interpretation and Comparison With Prior Work

Our study found no significant difference in overall cancer risk between transgender and cisgender individuals, but a significantly higher risk of precancerous lesions in transgender populations. While some previous studies suggest elevated cancer risks in transgender individuals, particularly hormone-sensitive cancers or those linked to infections such as HPV or HIV, our findings diverge from this [11]. Several factors may explain this discrepancy. First, our transgender cohort was relatively young (mean age=25.1, SD=14.0 years), whereas cancer risk increases substantially after age 50 [45]. Second, the small number of cancer cases limited statistical power. Third, disparities in health care access likely contributed to underdiagnosis. We found transgender individuals had fewer outpatient and inpatient visits compared with cisgender individuals at baseline, possibly reducing early detection opportunities. This aligns with previous research documenting lower health care usage and cancer screening rates among transgender individuals, potentially resulting in delayed diagnoses or underreported cases [46,47].

The elevated precancer risks observed in transgender individuals and subgroups align with recent findings. A JAMA Oncology review similarly reported higher HPV-related precancer rates among transgender individuals despite comparable cancer incidence, suggesting underlying vulnerabilities not fully captured by cancer diagnoses alone [10]. This suggests that while age may offer temporary protection against overt cancer, early-stage carcinogenic processes are already underway in this population. Biological factors such as prolonged hormone therapy, coupled with higher rates of smoking, alcohol use, and HIV, may accelerate precancerous changes at younger ages. Specifically, higher HPV-related precancer in MTF individuals likely reflects limited HPV screening and vaccination and greater HPV exposure [10]. Elevated colorectal precancer in both transgender subgroups could result from lower colorectal cancer screening uptake and health care engagement compared with cisgender populations [10]. These disparities underscore the need for targeted preventive care and screening interventions to mitigate cancer risk in transgender communities. Subgroup analyses comparing FTM to cisgender women and MTF to cisgender men revealed patterns consistent with the overall findings. While these associations remained significant after adjustment, the study was not powered to explore mechanistic differences. Detailed data on hormone therapy or other gender-affirming interventions were not available, limiting our ability to evaluate their potential influence on these observed disparities.

Our analysis also revealed several risk factors associated with precancer risk among transgender individuals, revealing subtle differences in disparities. For instance, transgender individuals with private insurance or Medicare exhibited lower precancer risks compared with those with Medicaid. Transgender individuals with Medicaid insurance may face increased risks due to limited access to specialized care or preventative services. For example, prior research indicates that Medicaid enrollees had lower rates of breast, cervical, and colorectal cancer screening than those with commercial insurance [48]. In addition, NHB transgender individuals demonstrated elevated precancer risks compared with their NHW counterparts, possibly due to systemic inequalities in health care access and usage, as well as higher rates of underlying health conditions in NHB individuals. According to the National Transgender Discrimination Survey, Black transgender adults in the United States have higher rates of unemployment, poverty, sexual assault, negative experiences with health care providers, and HIV than cisgender Black people [49]. Furthermore, the presence of substantial comorbidities emerged as a significant predictor of precancer risk among transgender individuals, reflecting the complex interplay between health status and cancer susceptibility. For example, conditions such as HIV, which are often prevalent in transgender populations, can weaken immune function and contribute to precancerous lesions. To tackle these differences, specific actions are needed, including improving the availability of specialized care and preventative services in Medicaid coverage for transgender individuals, implementing culturally competent health care practices within NHB communities, and addressing underlying health issues to reduce precancer risk.

### Strengths and Limitations

Our study has several strengths, including the use of comprehensive EHR data, which enabled a large-scale examination of cancer and precancer conditions among transgender individuals. The inclusion of matched cisgender cohorts enhances the validity of comparative analysis. However, our study also has limitations. First, the young age of our cohort limits generalizability to older transgender individuals, likely underestimating true cancer risks. Second, sample sizes for specific cancers were small, which limited the statistical power for subgroup analyses. Third, although health care usage measures were adjusted for in the analyses, residual confounding due to disparities in health care access and screening might persist. Fourth, our study may be influenced by residual confounding factors not fully captured in the EHR data, including socioeconomic status (eg, income and education), lifestyle behaviors (eg, smoking pack-years, alcohol use, and physical activity), sexual practices, environmental exposures, and hormone therapy use. Many of these factors are known to influence both cancer and precancer risk through complex, interrelated mechanisms. For example, limited financial resources and poor access to care may delay preventive screenings, while chronic stress or poor nutrition may increase biological vulnerability. Risky behaviors such as heavy alcohol use or tobacco smoking may also exert direct carcinogenic effects. Although EHRs may capture broad categories for some of these factors, they often lack granularity in terms of intensity, frequency, or timing, which limits our ability to adjust for their full impact in the analysis.

### Future Directions

Future research should include older and more diverse transgender populations and conduct age-stratified analyses to better assess lifetime cancer risk. Studies integrating EHRs with detailed behavioral, lifestyle, and hormone-related data are needed to improve risk estimation. Advanced analytic methods, such as causal inference or instrumental variable approaches, may also help address unmeasured confounding.

### Conclusion

This study offers important insights into cancer and precancer burden among transgender individuals and underscores the need for targeted interventions to reduce cancer disparities in this underserved population.

## Supplementary material

10.2196/73843 Multimedia Appendix 1 Transgender diagnosis codes and keywords.

10.2196/73843 Multimedia Appendix 2 Cancer sites and corresponding precancer conditions.

10.2196/73843 Checklist 1 STROBE-RECORD checklist.

## Abbreviations

CCI: Charlson Comorbidity Index
EHR: electronic health record
FTM: female-to-male
HIV: human immunodeficiency virus
HPV: human papillomavirus
*ICD*: *International Classification of Diseases*
IDR: Integrated Data Repository
MTF: male-to-female
NHB: non-Hispanic Black
NHW: non-Hispanic White
RECORD: REporting of studies Conducted using Observational Routinely-collected Data
sHR: subdistribution hazard ratio
STROBE: Strengthening the Reporting of Observational Studies in Epidemiology
UF: University of Florida
