# Supplementary material for: The Burden of Cancer and Precancerous Conditions Among Transgender Individuals in a Large Health Care Network: Retrospective Cohort Study
Source: JMIR Cancer. 2025 Sep 8;11:e73843. doi: 10.2196/73843 (PMC12416876; doi:10.2196/73843)
Supplement: Multimedia Appendix 1 [file cancer-v11-e73843-s001.docx]

| **Transgender diagnosis code** | |  |
| --- | --- | --- |
|  | Code | Concept |
| ICD-9 | 302.3 | Transvestic fetishism |
|  | 302.5 | Trans-sexualism |
|  | 302.50 | Trans-sexualism with unspecified sexual history |
|  | 302.51 | Trans-sexualism with an asexual history |
|  | 302.52 | Trans-sexualism with homosexual history |
|  | 302.53 | Trans-sexualism with heterosexual history |
|  | 302.6 | Gender identity disorder in children |
|  | 302.85 | Gender identity disorder in adolescents or adults |
| ICD-10 | F64 | Gender identity disorders |
|  | F64.0 | Transsexualism |
|  | F64.1 | Dual role transvestism |
|  | F64.2 | Gender identity disorder of childhood |
|  | F64.8 | Other gender identity disorders |
|  | F64.9 | Gender identity disorder, unspecified |
|  | F65.1 | Transvestic fetishism |
|  | Z87.890 | Personal history of sex reassignment |
| **Transgender keyword** |  |  |
|  | **root word** | **regex** |
|  | transgender | (?i)(\W\|^)trans([ \t]+\|\r+\|\n+\|\r\n+\|\n\r+\|\-\|)gender(\W\|$) |
|  | transsex | (?i)(\W\|^)trans([ \t]+\|\r+\|\n+\|\r\n+\|\n\r+\|\-\|)sex(\W\|$) |
|  |  | (?i)(\W\|^)transex(\W\|$) |
|  | transsexual | (?i)(\W\|^)trans([ \t]+\|\r+\|\n+\|\r\n+\|\n\r+\|\-\|)sexual(\W\|$) |
|  |  | (?i)(\W\|^)transexual(\W\|$) |
|  | transsexuality | (?i)(\W\|^)trans([ \t]+\|\r+\|\n+\|\r\n+\|\n\r+\|\-\|)sexuality(\W\|$) |
|  |  | (?i)(\W\|^)transexuality(\W\|$) |
|  | male to female | (?i)(\W\|^)male([ \t]+\|\r+\|\n+\|\r\n+\|\n\r+\|\-\|)to([ \t]+\|\r+\|\n+\|\r\n+\|\n\r+\|\-\|)female(\W\|$) |
|  | female to male | (?i)(\W\|^)female([ \t]+\|\r+\|\n+\|\r\n+\|\n\r+\|\-\|)to([ \t]+\|\r+\|\n+\|\r\n+\|\n\r+\|\-\|)male(\W\|$) |
|  | intersex | (?i)(\W\|^)inter([ \t]+\|\r+\|\n+\|\r\n+\|\n\r+\|\-\|)sex(\W\|$) |
|  | intersexual | (?i)(\W\|^)inter([ \t]+\|\r+\|\n+\|\r\n+\|\n\r+\|\-\|)sexual(\W\|$) |
|  | dual gender | (?i)(\W\|^)dual([ \t]+\|\r+\|\n+\|\r\n+\|\n\r+\|\-\|)gender(\W\|$) |
|  | trans women | (?i)(\W\|^)trans([ \t]+\|\r+\|\n+\|\r\n+\|\n\r+\|\-\|)women(\W\|$) |
|  | trans men | (?i)(\W\|^)trans([ \t]+\|\r+\|\n+\|\r\n+\|\n\r+\|\-\|)men(\W\|$) |
|  | trans female | (?i)(\W\|^)trans([ \t]+\|\r+\|\n+\|\r\n+\|\n\r+\|\-\|)female(\W\|$) |
|  | trans male | (?i)(\W\|^)trans([ \t]+\|\r+\|\n+\|\r\n+\|\n\r+\|\-\|)male(\W\|$) |
|  | trans people | (?i)(\W\|^)trans([ \t]+\|\r+\|\n+\|\r\n+\|\n\r+\|\-\|)people(\W\|$) |
|  | agender | (?i)(\W\|^)agender(\W\|$) |
|  | bigender | (?i)(\W\|^)bi([ \t]+\|\r+\|\n+\|\r\n+\|\n\r+\|\-\|)gender(\W\|$) |
|  | demimale | (?i)(\W\|^)demi([ \t]+\|\r+\|\n+\|\r\n+\|\n\r+\|\-\|)male(\W\|$) |
|  | demifemale | (?i)(\W\|^)demi([ \t]+\|\r+\|\n+\|\r\n+\|\n\r+\|\-\|)female(\W\|$) |
|  | nonbinary | (?i)(\W\|^)non([ \t]+\|\r+\|\n+\|\r\n+\|\n\r+\|\-\|)binary(\W\|$) |
|  | gender non-conforming | (?i)(\W\|^)gender([ \t]+\|\r+\|\n+\|\r\n+\|\n\r+\|\-\|)non([ \t]+\|\r+\|\n+\|\r\n+\|\n\r+\|\-\|)conforming(\W\|$) |
|  | gender nonconformity | (?i)(\W\|^)gender([ \t]+\|\r+\|\n+\|\r\n+\|\n\r+\|\-\|)non([ \t]+\|\r+\|\n+\|\r\n+\|\n\r+\|\-\|)conformity(\W\|$) |
|  | neutrois | (?i)(\W\|^)neutrois(\W\|$) |
|  | two spirit | (?i)(\W\|^)two([ \t]+\|\r+\|\n+\|\r\n+\|\n\r+\|\-\|)spirit(\W\|$) |
|  | gender variant | (?i)(\W\|^)gender([ \t]+\|\r+\|\n+\|\r\n+\|\n\r+\|\-\|)variant(\W\|$) |
|  | genderfluid | (?i)(\W\|^)gender([ \t]+\|\r+\|\n+\|\r\n+\|\n\r+\|\-\|)fluid(\W\|$) |
|  | genderqueer | (?i)(\W\|^)gender([ \t]+\|\r+\|\n+\|\r\n+\|\n\r+\|\-\|)queer(\W\|$) |
|  | preferred pronoun | (?i)(\W\|^)preferred([ \t]+\|\r+\|\n+\|\r\n+\|\n\r+\|\-\|)pronoun(\W\|$) |
|  | preferred gender pronoun | (?i)(\W\|^)preferred([ \t]+\|\r+\|\n+\|\r\n+\|\n\r+\|\-\|)gender([ \t]+\|\r+\|\n+\|\r\n+\|\n\r+\|\-\|)pronoun(\W\|$) |
|  | gender dysphoria | (?i)(\W\|^)gender([ \t]+\|\r+\|\n+\|\r\n+\|\n\r+\|\-\|)dysphoria(\W\|$) |
|  | gender disorder | (?i)(\W\|^)gender([ \t]+\|\r+\|\n+\|\r\n+\|\n\r+\|\-\|)disorder(\W\|$) |
|  | gender incongruence | (?i)(\W\|^)gender([ \t]+\|\r+\|\n+\|\r\n+\|\n\r+\|\-\|)incongruence(\W\|$) |
|  | gender identity dysphoria | (?i)(\W\|^)gender([ \t]+\|\r+\|\n+\|\r\n+\|\n\r+\|\-\|)identity([ \t]+\|\r+\|\n+\|\r\n+\|\n\r+\|\-\|)dysphoria(\W\|$) |
|  | gender identity disorder | (?i)(\W\|^)gender([ \t]+\|\r+\|\n+\|\r\n+\|\n\r+\|\-\|)identity([ \t]+\|\r+\|\n+\|\r\n+\|\n\r+\|\-\|)disorder(\W\|$) |
|  | gender affirming surgery | (?i)(\W\|^)gender([ \t]+\|\r+\|\n+\|\r\n+\|\n\r+\|\-\|)affirming([ \t]+\|\r+\|\n+\|\r\n+\|\n\r+\|\-\|)surgery(\W\|$) |
|  | gender affirmation surgery | (?i)(\W\|^)gender([ \t]+\|\r+\|\n+\|\r\n+\|\n\r+\|\-\|)affirmation([ \t]+\|\r+\|\n+\|\r\n+\|\n\r+\|\-\|)surgery(\W\|$) |
|  | transgender surgery | (?i)(\W\|^)transgender([ \t]+\|\r+\|\n+\|\r\n+\|\n\r+\|\-\|)surgery(\W\|$) |
|  | sex reassignment | (?i)(\W\|^)sex([ \t]+\|\r+\|\n+\|\r\n+\|\n\r+\|\-\|)reassignment(\W\|$) |
|  | gender reassignment | (?i)(\W\|^)gender([ \t]+\|\r+\|\n+\|\r\n+\|\n\r+\|\-\|)reassignment(\W\|$) |
|  | sex reassignment surgery | (?i)(\W\|^)sex([ \t]+\|\r+\|\n+\|\r\n+\|\n\r+\|\-\|)reassignment([ \t]+\|\r+\|\n+\|\r\n+\|\n\r+\|\-\|)surgery(\W\|$) |
|  | gender reassignment surgery | (?i)(\W\|^)gender([ \t]+\|\r+\|\n+\|\r\n+\|\n\r+\|\-\|)reassignment([ \t]+\|\r+\|\n+\|\r\n+\|\n\r+\|\-\|)surgery(\W\|$) |
|  | sex change | (?i)(\W\|^)sex([ \t]+\|\r+\|\n+\|\r\n+\|\n\r+\|\-\|)change(\W\|$) |
|  | cross sex | (?i)(\W\|^)cross([ \t]+\|\r+\|\n+\|\r\n+\|\n\r+\|\-\|)sex(\W\|$) |
|  | cross gender | (?i)(\W\|^)cross([ \t]+\|\r+\|\n+\|\r\n+\|\n\r+\|\-\|)gender(\W\|$) |
|  | transvest | (?i)(\W\|^)transvest(\W\|$) |
|  | transvestite | (?i)(\W\|^)transvestite(\W\|$) |
