# Supplementary material for: The Burden of Cancer and Precancerous Conditions Among Transgender Individuals in a Large Health Care Network: Retrospective Cohort Study
Source: JMIR Cancer. 2025 Sep 8;11:e73843. doi: 10.2196/73843 (PMC12416876; doi:10.2196/73843)
Supplement: Multimedia Appendix 2 [file cancer-v11-e73843-s002.docx]

| Cancer site | Precancer | ICD codes |
| --- | --- | --- |
| Oropharyngeal, Head and neck cancer [1] | Leukoplakia; Erythroplakia;Oral submucosal fibrosis; Oral lichen planus; Actinic keratosis | 528, K13.2, K13.7, 697.0, K13.5, L43, D50.1, 280.8,  702.0, L57.0 |
| Colorectal | Familial adenomatous polyposis; Tubular adenomas; Villous adenomas; Tubulovillous adenomas; Hyperplastic polyposis; Cowden syndrome; Lynch syndrome; Peutz-Jeghers syndrome; Juvenile polyposis syndrome; Chronic Inflammatory Bowel Disease | 211, 238, 555-558, 759, D12, D48, K50-K52, Q85.8, K55.9, Z15.09, V84.09, K63.5, K62.1 |
| Lung [2] | Squamous dysplasia/carcinoma in situ; Atypical adenomatous hyperplasia; Diffuse idiopathic pulmonary neuroendocrine cell hyperplasia; | 227.3, 233.2, D02.2, D48.1, J84.10, D38.1 |
| Stomach | Gastric epithelial dysplasia; Gastric adenoma; Intestinal metaplasia; Chronic atrophic gastritis, Familial adenomatous polyposis, | 230, D00, K31, D13.1, K29.4, 535.1, B96.81, 041.86 |
| Esophagus | Barrett's esophagus; | 530.8, K22.7 |
| Bladder [3] | Carcinoma in situ (also called CIS or Tis); Urothelial hyperplasia; Urothelial papilloma; Urothelial dysplasia; Intestinal metaplasia; Keratinising squamous metaplasia; Condyloma acuminatum | 223.3, 233.7, 595.8, 596.8, D09.0, D30, N30-N32 |
| Liver [4] | Dysplastic nodules (DN); dysplastic foci (DF) | 571.5, D48.0, K74.60 |
| Kidney [5] [6] | Von Hippel-Lindau syndrome; Adenoma; Nephroblastomatosis; Intratubular epithelial dysplasia; High or low-grade renal intratubular neoplasia | 759.6, D30, Q85.01, 211.3 |
| Pancreas | Mucinous cystic neoplasm (MCN); Intraductal papillary mucinous neoplasm (IPMN); Solid pseudopapillary neoplasm (SPN); Neuroendocrine tumors; | 211.6, D13.6, 577, K85, K86* |
| Breast [7] | Atypical hyperplasia; Ductal carcinoma in situ (DCIS); Lobular carcinoma in situ; | 233, D05 |
| Cervical | Cervical intraepithelial neoplasia [CIN], grade I Low grade squamous intraepithelial lesion (LSIL); CIN, grade II; CIN III; | 233.1, 622.1, D06, N87 |
| Vaginal | Vaginal Intraepithelial Neoplasia 1 (VAIN 1); VAIN 2; VAIN 3 | 233.31, 623.0, N89 |
| Vulva | Vulvar Intraepithelial Neoplasia 1 (VIN 1); VIN 2; VIN 3 | 233.32, 624, D07.1, N90 |
| Anal | Low-grade squamous cell intraepithelial lesions (LSILs) - anal intraepithelial neoplasia (AIN 1); HSIL - AIN 2; HSIL- AIN 3 | 230, 569.44, D01.3, K62.83,230.5, 230.6 |
| Penis [8] | Penile intraepithelial neoplasia (PeIN); Balanitis xerotica obliterans (BXO); Buschke-Lowenstein tumour bowenoid papulosis; Leukoplakia | 078.11, 233.5, 607.81, 607.0, 701.1, A63.0, D07.4, L90.0, N48.0 |

Reference:

1. Yardimci G, Kutlubay Z, Engin B, Tuzun Y. Precancerous lesions of oral mucosa. World J Clin Cases Baishideng Publishing Group Inc.; 2014 Dec 16;2(12):866–872. PMID:25516862

2. Greenberg AK, Yee H, Rom WN. Preneoplastic lesions of the lung. Respir Res Springer Science and Business Media LLC; 2002 Apr 4;3(1):20. PMID:11980589

3. Samaratunga H, Martignoni G, Egevad L, Delahunt B. Premalignant lesions of the urinary bladder. Pathology 2013 Apr;45(3):243–250. PMID:23448810

4. Niu Z-S, Niu X-J, Wang W-H, Zhao J. Latest developments in precancerous lesions of hepatocellular carcinoma. World J Gastroenterol Baishideng Publishing Group Inc.; 2016 Mar 28;22(12):3305–3314. PMID:27022212

5. Wan X, Xing Z, Ouyang J, Liu H, Cheng C, Luo T, Yu S, Meihua L, Huang S. Histomorphological and ultrastructural cadmium-induced kidney injuries and precancerous lesions in rats and screening for biomarkers. Biosci Rep Portland Press Ltd.; 2022 Jun 30;42(6). PMID:35678542

6. Haas NB, Nathanson KL. Hereditary kidney cancer syndromes. Adv Chronic Kidney Dis Elsevier BV; 2014 Jan;21(1):81–90. PMID:24359990

7. Lebeau A. Precancerous lesions of the breast. Breast Care (Basel) S. Karger AG; 2010 Aug;5(4):204–206. PMID:22590439

8. Flagg EW, Schwartz R, Weinstock H. Prevalence of anogenital warts among participants in private health plans in the United States, 2003-2010: potential impact of human papillomavirus vaccination. Am J Public Health American Public Health Association; 2013 Aug;103(8):1428–1435. PMID:23763409
