# Supplementary material for: The Burden of Cancer and Precancerous Conditions Among Transgender Individuals in a Large Health Care Network: Retrospective Cohort Study
Source: JMIR Cancer. 2025 Sep 8;11:e73843. doi: 10.2196/73843 (PMC12416876; doi:10.2196/73843)
Supplement: Checklist 1 [file cancer-v11-e73843-s003.docx]

| Checklist item | Page no |
| --- | --- |
| STROBE 1a: Study design in title/abstract | 1 |
| STROBE 1b: Informative abstract | 1t |
| STROBE 2-3: Background & objectives | 2-3 |
| STROBE 4: Study design details | 3-4 |
| STROBE 5: Setting, dates, location | 3-4 |
| STROBE 6a: Eligibility, cases/controls | 3-4 |
| STROBE 6b: Matching criteria, # of controls | 3-4 (1:10:10) |
| STROBE 7: Variables defined | 4-5 |
| STROBE 8: Data sources & measurements | 3 |
| STROBE 9: Bias | Partially addressed |
| STROBE 10: Study size calculation | X |
| STROBE 11: Quantitative variables handling | 4-5 |
| STROBE 12: Statistical methods | 5-6 |
| STROBE 13: Flow diagram | X |
| STROBE 14a: Participant characteristics | 7 |
| STROBE 14b: Missing data reporting | 5-6 |
| STROBE 15: Outcome data | 8-10 |
| STROBE 16: Main results with CIs | 10 |
| STROBE 17: Other analyses | 11-12 (subgroups and interactions) |
| STROBE 18-20: Discussion, limitations, interpretation | 12-15 |
| STROBE 21: Generalizability | X |
| STROBE 22: Funding | 16 |
| RECORD 1.1: Data type in title/abstract | 1 |
| RECORD 6.1: Study population selection methods | 3-4 |
| RECORD 6.2: References for codes/algorithms | 3-4, Multimedia Appendix |
| RECORD 6.3: Validation of codes/algorithms | 3-4 (precision, recall, and *F*_1_) |
| RECORD 7.1: Complete list of codes/classifications | X |
| RECORD 12.1: Data linkage (if applicable) | X |
| RECORD 12.2: Data quality assessment | X |
| RECORD 13.1: Flow diagram | X |
